# Assessment of immune responses to *Brucella abortus* S19 vaccination in cattle and buffaloes

**DOI:** 10.3389/fimmu.2025.1663259

**Published:** 2025-11-10

**Authors:** Rajeswari Shome, Prabhakar Yallanur Konda, Somy Skariah, Gandu Shanmugam, Praveen Kumar Attiganahalli Muninarayanaswamy, Pavan Kalyan Nagaraja, Kirti Megha, Nanditha Thimmappa Rajeshwari, Nagalingam Mohandoss

**Affiliations:** Indian Council of Agricultural Research (ICAR)-National Institute of Veterinary Epidemiology and Disease Informatics, Yelahanka, Bengaluru, India

**Keywords:** brucellosis, *Brucella abortus* S19, cell-mediated immunity, humoral immunity, innate immunity

## Abstract

**Introduction:**

The *Brucella abortus* S19 vaccine remains the global reference standard for bovine brucellosis prevention. This study comprehensively evaluated the safety and immunogenicity of S19 at a dose of 4 × 10^9^ colony-forming units (CFU) in cattle and buffalo calves.

**Methods:**

A total of 180 female calves (90 cattle and 90 buffalo calves) aged 4–8 months were equally distributed across six postvaccination intervals (15 animals/group/species for days postvaccination [DPV] intervals: DPV < 21, 21–45, 46–60, 61–90, 91–120, and > 120 days). All calves were confirmed seronegative for anti-*Brucella* antibodies by Rose Bengal Plate Test (RBPT) and indirect enzyme-linked immunosorbent assay (ELISA) prior to vaccination. Vaccinated animals were monitored for 3 weeks for adverse effects. Humoral immunity was assessed using RBPT and Serum Agglutination Test (SAT); antibody isotypes immunoglobulin M (IgM)/immunoglobulin G (IgG) were quantified by indirect ELISA, cytokines tumor necrosis factor-α (TNF-α), interleukin (IL)-6, IL-8, and IL-10 were measured by competitive inhibition ELISA, while IL-12, IL-1β, and interferon gamma (IFN-γ) were quantified using high-sensitivity sandwich ELISAs. All samples were processed by cold centrifugation (4°C) and stored at − 20°C until analysis.

**Results:**

No local/systemic reactions were observed, confirming safety. Significant humoral responses (*p* < 0.05) peaked at DPV interval < 21 (SAT: 640 IU/mL), declining gradually but persisting beyond 120 DPV. Cattle calves showed faster IgM-to-IgG switching (*p* < 0.05), with peak IgM (179.2 ± 5.5 *vs*. 163.7 ± 5.9) and IgG (220.6 ± 13.9 *vs*. 187.5 ± 9.5) in cattle versus buffalo calves. Proinflammatory cytokines (TNF-α, IL-6, IL-8, IL-12, IL-1β) and IL-10 peaked at DPV interval 46–60 (*p* < 0.05). IFN-γ (Th1 marker) peaked earlier, at DPV interval 21–45 (cattle 307.7 pg/mL ± 13.4 pg/mL *vs*. buffalo 274.2 pg/mL ± 11.7 pg/mL, *p* < 0.05), indicating robust CMI. All comparisons were performed using one-way ANOVA (GraphPad Prism 10), with significance set at *p* < 0.05.

**Conclusion:**

*B. abortus* S19 at 4 × 10^9^ CFU/dose safely induces durable humoral and cellular immunity in both species, with cattle mounting faster adaptive responses than buffaloes. The comprehensive immune profile supports its use as an effective vaccination strategy.

## 1 Introduction

Bovine brucellosis, caused by *Brucella abortus*, represents a major global zoonotic threat with significant economic and public health consequences (1). As one of the most widespread bacterial infections affecting livestock, it causes substantial reproductive losses, including abortions (10%–15% in cattle, 5%–10% in buffaloes) and reduced milk production. The disease also poses serious zoonotic risks through occupational exposure and the consumption of contaminated dairy products (2). The *B. abortus* S19 vaccine has long been the cornerstone of brucellosis control programs, yet its effectiveness is limited by two critical limitations: residual virulence in vaccinated animals and interference with standard serodiagnostic tests (3).

The pathogenesis of *B. abortus* infection involves a complex interplay between microbial virulence factors and host immune defense mechanisms. Following mucosal entry, the pathogen evades phagocytic clearance through its unique O-antigen and lipopolysaccharide (LPS) structure, effectively inhibiting dendritic cell maturation (4, 5). Intracellular survival is mediated by the VirB type IV secretion system, which suppresses critical proinflammatory cytokines, including tumor necrosis factor-α (TNF-α) and interferon gamma (IFN-γ) (6). Of particular clinical significance is the bacterium’s tropism for placental tissue, where erythritol acts as a potent growth stimulant, leading to severe placentitis and abortion (7). Recent comparative studies have revealed fundamental differences in immune responses between cattle and buffaloes, with buffaloes exhibiting weaker T helper cell type 1 (Th1)-mediated responses and consequently delayed bacterial clearance (8). Significant immunological disparities between the two species have been well documented in brucellosis infection.

Diagnostic tests such as the Rose Bengal plate test (RBPT) and serum agglutination test (SAT) demonstrate higher baseline antibody titers in buffaloes during early infection, although both species maintain persistent immunoglobulin M (IgM) and immunoglobulin G (IgG) responses (9). Buffaloes exhibit delayed IgG class switching and prolonged IgM dominance, correlating with their weaker bacterial clearance capacity (10). At the cytokine level, cattle typically mount robust Th1-polarized responses characterized by elevated TNF-α, interleukin (IL)-12, and IFN-γ, whereas buffaloes show blunted IFN-γ production coupled with elevated IL-6 and IL-10 (11). Both species demonstrate early IL-8 and IL-1β surges, although buffaloes maintain chronically elevated IL-1β levels, which may contribute to placental pathology. These immunological differences underscore species-specific approaches to both diagnosis and vaccination (12–14).

*B. abortus* S19 vaccine serves as the cornerstone of calfhood vaccination programs; however, critical knowledge gaps persist regarding India’s standardized *B. abortus* S19 vaccine at a formulation of 4 × 10^9^ (CFU)/dose, which have been underrepresented in immunological studies. This investigation represents the first comprehensive evaluation of this vaccine formulation in both cattle and buffalo calves, employing a longitudinal study design with multiple postvaccination intervals ranging from < 21 to > 120 days postvaccination (DPV) to systematically assess safety, immunogenicity, and duration of protection under field conditions. We aimed to (1): validate the safety profile of the 4 × 10^9^ CFU/dose in both species (2); characterize early immune biomarkers (IgM, IL-8, IL-1β) predictive of vaccine efficacy (3); determine the durability of protective immunity beyond 120 DPV; and (4) establish species-specific immune response patterns to optimize vaccination strategies. Our findings provide crucial evidence supporting India’s brucellosis control program while advancing veterinary vaccinology through the identification of key immunological correlates of protection in diverse bovine populations.

## 2 Materials and methods

### 2.1 Experimental layout

For the study, five villages with more than 250 dairy animals, each located approximately 2 km apart within the same region, were selected in Bengaluru Rural District, Karnataka, India, during 2023 (15). The climatic conditions, feed and fodder, husbandry practices, and healthcare support for the animals were similar across villages, despite the animals being owned by different farmers. The cattle and buffalo breeds selected were Holstein Friesian, Jersey, and Murrah. The farm owners and the consultant veterinarians were informed about the study, and the consented animals were chosen for pre- and postvaccination blood sample collection. The calves were dewormed 1 week before vaccination and fed according to the farmer’s choice of feed, fodder, and water. A total of 90 female cattle and buffalo calves, aged 4–8 months, were equally distributed across six postvaccination intervals (15 animals/group species for DPV < 21, 21–45, 46–60, 61–90, 91–120, and > 120 days). All calves were serologically screened for anti-*Brucella* antibodies using both RBPT and SAT, and (2) an indirect protein G-based enzyme-linked immunosorbent assay (iELISA) during a 3-week prevaccination period to confirm seronegative status (16). The cattle and buffalo calves received *B. abortus* S19 vaccine at a dose of 4 × 10^9^CFU/dose, according to the vaccination program in the farmer’s household. All animals were ear-tagged according to the national registry, allowing for easy identification. The health status, vaccination procedures, and blood sampling of the vaccinated calves were monitored under established ethical protocols by a veterinarian in field conditions.

### 2.2 Vaccination study

The *B. abortus* S19 freeze-dried live vaccine, developed according to the Indian Pharmacopoeia (IP) guidelines and currently utilized in India for the prophylaxis of brucellosis in female cattle and buffalo calves, was employed in this study. The 10-dose vaccine vial was reconstituted with diluent according to the manufacturer’s instructions, and 2.0 mL of vaccine, consisting of 4 × 10^9^ CFU/dose, was administered via subcutaneous injection into the cervical region of the calves. The vaccinated calves were carefully monitored for 48–72-h postvaccination for potential local reactions, including pain, swelling, redness, and allergic reaction, as well as systemic reactions such as anorexia, dullness, depression, and fever, up to 21 days postvaccination (16).

### 2.3 Evaluation of various immune responses

Blood samples were collected aseptically from the jugular vein into sterile vacutainers without anticoagulant and transported within 4-6-h to prevent degradation of TNF-α, IL-6, IL-8, IL-10, IL-1β, and IL-12. Serum was separated from the blood samples by centrifugation at 3,000 rpm for 3–5 min and stored at − 20°C until analysis to preserve analyte integrity. Adherence to cold chain protocols minimized cytokine degradation, ensuring assay reliability.

### 2.4 Evaluation of humoral immune response using serological assays

The humoral immune response was evaluated using the RBPT, SAT, and iELISA. In the RBPT, agglutination reactions were scored as strong positive (+++) if visible within 10 s, moderate positive (++) at 30 s, or mild positive (+) at 60 s, while no agglutination was recorded as negative (−). For the SAT, the endpoint titer was defined as the highest serum dilution showing 50% agglutination, with titers ≥ 1:80 (160 IU/mL) considered significant for vaccinated animals. The antigens used for both RBPT and SAT were procured from the Institute of Animal Health and Veterinary Biologicals (IAH and VB), Bengaluru, India. These antigens were prepared using the *B. abortus* strain S99, the internationally recognized reference strain for serological testing of *Brucella* infections in animals and humans. Strain S99 is a smooth, virulent strain with stable antigenic properties and is routinely used in the standardization and production of diagnostic reagents for brucellosis surveillance and control programs.

### 2.5 Evaluation of IgG and IgM antibodies

To assess the humoral immune response, *B. abortus* smooth lipopolysaccharide (sLPS)-specific IgM and IgG antibodies were measured using an indirect ELISA. Briefly, 96-well polysorb microtiter plates were coated with *B. abortus* sLPS antigen (100 ng/well) and incubated overnight at 37°C. After blocking with 2% correct it to bovine gelatin prepared with 0.05% Tween-20 in phosphate-buffered saline (PBST) for 1 h at 37°C, diluted serum samples (1:100 in blocking buffer) were added and incubated for 1 h at 37°C. For IgM detection, serum samples were pretreated with protein G to eliminate IgG interference. Following PBST washes, IgM-specific antibodies were detected using an antibovine IgM-Horseradish peroxidase (HRP) conjugate at a 1:6,000 dilution (Sigma-Aldrich, St. Louis, MO, USA), while IgG-specific antibodies were detected directly using a protein G-HRP conjugate at a 1:8,000 dilution (due to its high affinity for IgG). After 1 h of incubation at 37°C and subsequent washes, the reaction was developed using a substrate solution containing 5 mg of *O*-phenylenediamine dihydrochloride (OPD) and 50 µL of 3% H_2_O_2_ in 12 mL of distilled water. The reaction was stopped with 1 M H_2_SO_4_, and absorbance was measured at 492 nm. Antibody levels were quantified against a standard curve generated from reference sera, with results expressed as optical density (OD) values converted into percent positivity (PP) values. The cutoff PP was categorized as negative (if PP < 25%) and positive (if PP > 25%).

### 2.6 Evaluation of the innate immune response

Bovine proinflammatory (TNF-α, IL-6, IL-8) and anti-inflammatory (IL-10) cytokines were quantified using a competitive inhibition enzyme immunoassay (cELISA) platform (CUSABIO, Wuhan, China), demonstrating the assay’s adaptability for multiplexed cytokine profiling. Briefly, microtiter plates precoated with goat antirabbit antibody were incubated with samples alongside HRP-conjugated and native (unlabeled) TNF-α, IL-6, IL-8, or IL-10, initiating competitive binding to their respective antibodies. The OD postreaction to substrate addition was recorded within 10 min using a microplate reader set at 450 nm. The colorimetric signals were interpreted as inversely proportional to cytokine concentrations. The assay’s dynamic ranges (TNF-α: 0.1–20 ng/mL; IL-6/IL-10: 5–1,000 pg/mL; IL-8: 50–2,000 pg/mL) allowed precise quantification across physiologically relevant concentrations.

The concentrations of IL-12 (Th1-polarizing cytokine) and IL-1β (proinflammatory mediator) were quantified using a high-sensitivity sandwich ELISA (CUSABIO, Wuhan, China), leveraging the assay’s dual-antibody system for enhanced specificity. Microtiter plates precoated with antibovine IL-12 or IL-1β antibodies captured target cytokines from samples, followed by sequential incubation with biotinylated detection antibodies and HRP-conjugated avidin. The OD of all samples was determined within 5 min using a microplate reader set at 450 nm. The enzymatic reaction generated colorimetric signals proportional to cytokine levels, with dynamic ranges optimized for bovine serum analysis (IL-12: 6.25–400 pg/mL; IL-1β: 62.5–4,000 pg/mL).

### 2.7 Evaluation of cell-mediated immunity response

Bovine IFN-γ, a critical mediator of cell-mediated immunity (CMI) and macrophage activation, was quantified using a high-sensitivity sandwich ELISA (CUSABIO, Wuhan, China). This method leverages a dual-antibody capture system for high specificity, with a precoated monoclonal antibody selectively immobilizing IFN-γ from serum samples. Following removal of unbound proteins, a biotinylated secondary antibody and streptavidin-HRP conjugate were added. The OD was recorded within 5 min, using a microplate reader set at 450 nm. The enzymatic colorimetric signal, linearly proportional to IFN-γ concentration across a broad dynamic range (31.25–2.000 pg/mL), enabled precise quantification of both basal and infection/vaccine-induced levels.

### 2.8 Statistical analysis and data quantification

All experimental data are expressed as mean ± standard deviation. Statistical comparisons of immune responses elicited by the *B. abortus* S19 vaccine were performed using one-way ANOVA in GraphPad Prism 10 with statistical significance set at *p* < 0.05. Qualitative RBPT results were quantified using a standardized scoring system: negative results (no agglutination) scored 0, mild (+) scored 5, moderate (++) scored 10, and strong (+++) agglutination scored 15 points, based on reaction time. SAT data were similarly converted to numerical scores according to endpoint serum dilution titers expressed in International Units per milliliter.

## 3 Results

### 3.1 Study population and vaccine safety assessment

The study included 90 female cattle and buffalo calves (15 calves/DPV intervals), all of which were confirmed negative for anti-*Brucella* antibodies prior to vaccination using RBPT and iELISA. The animals were vaccinated with *B. abortus* S19 at a dose of 4 × 10^9^ CFU and were maintained by farmers in their households. The vaccinated calves showed no pain, swelling, erythema, allergic reactions, or fever during the 2–3-day postvaccination monitoring period. All calves maintained normal health status and feeding behavior, demonstrating excellent vaccine tolerance and confirming the safety profile of the administered dose.

### 3.2 Humoral immune response profiling through serology

#### 3.2.1 Evaluation of humoral immune response using RBPT

**Figure 1:**
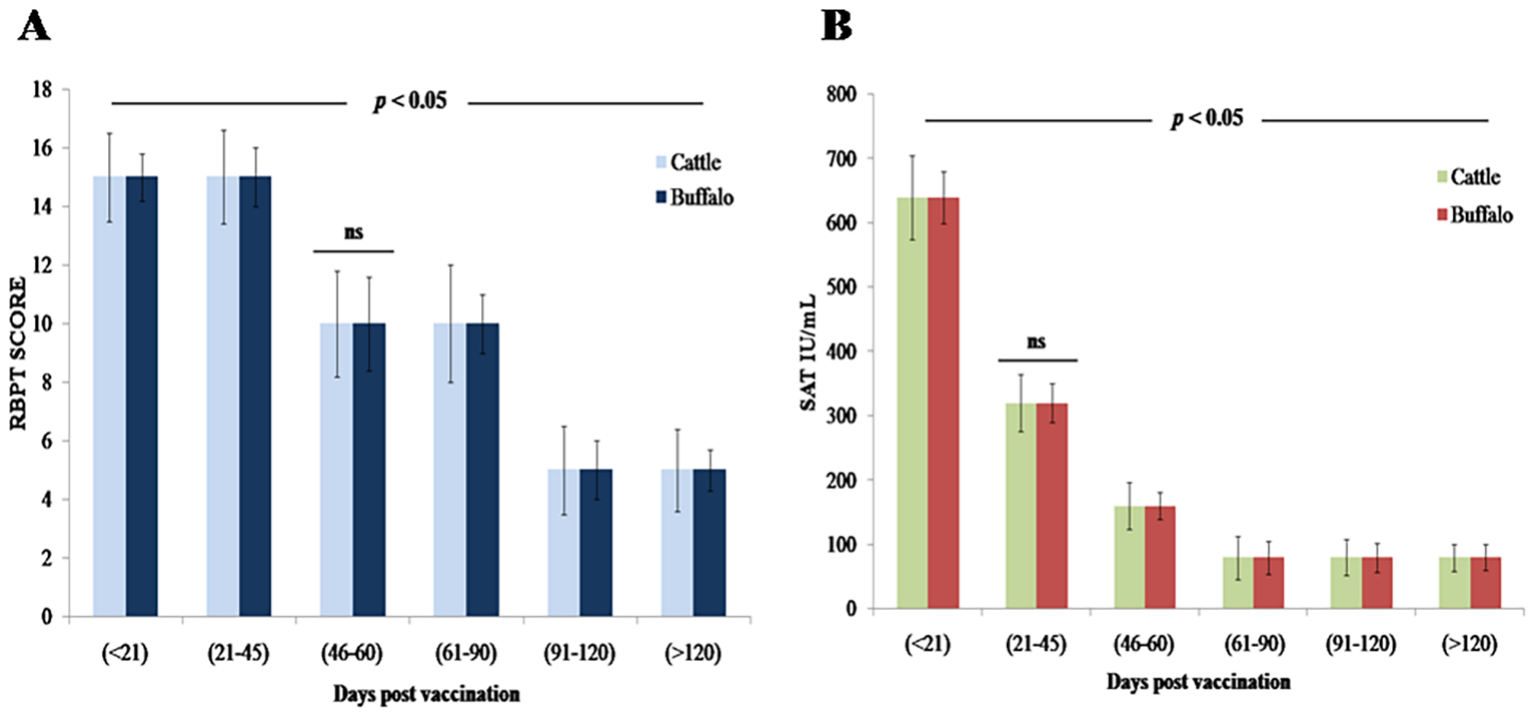
Antibody response kinetics in cattle and buffalo calves vaccinated with *B. abortus* S19 (4 × 10^9^ CFU/dose) at different DPV intervals: **(A)** RBPT **(B)** SAT titers. Data are presented as Mean ± SD; statistical difference of antibody titers.

A statistically significant increase in antibody levels was observed by RBPT following vaccination with *B. abortus* S19 at 4 × 10^9^ CFU/dose, with strong agglutination noted between DPV < 21 and 21–45 in both species, followed by a gradual decline (*p* < 0.05). Detectable agglutination persisted through DPV > 120, indicating a sustained immunological response, though no agglutination was observed beyond this period (**Figure 1A**, **Table 1**). No statistically significant differences were observed between cattle and buffalo calves (ns, *p* > 0.05), suggesting similar immune responses to vaccination.

**Table 1:** Humoral immune response profile in both cattle and buffalo calves vaccinated with *B. abortus* S19 (4 × 10^9^ CFU/dose), as assessed by RBPT at different DPV intervals.

DPVs	*B. abortus* S19 in cattle and buffalo calves (4 × 10^9^ CFU/dose; *n* = 15)			
	−	+	++	+++
0	15			
< 21				15
21–45				15
46–60			15	
61–90			15	
91–120		15		
> 120		15		

Data are presented as mean ± SD.

*RBPT*, Rose Bengal Plate Test; *B. abortus S19*, *Brucella abortus* S19 strain vaccine; DPV, days of postvaccination; C*FU*, colony forming units; (−) negative; (+) mild positive; (++) moderate positive; (+++) strong positive.

#### 3.2.2 Evaluation of humoral immune response using SAT

The SAT analysis revealed statistically significant (*p* < 0.01) serum antibody titers of 640 IU/mL (DPV < 21) and 320 IU/mL (DPV 21–45) in both cattle and buffalo calves vaccinated with *B. abortus* S19 at 4 × 10^9^ CFU/dose (**Figure 1B**). Antibody levels gradually but significantly decline (*p* < 0.05) from DPV 46–60 through 91–120, yet remained detectable (≥ 80 IU/mL) through DPV > 120. No significant agglutination (*p* > 0.05) was observed beyond DPV 120, confirming eventual antibody waning (**Table 2**). Throughout the study period, no statistically significant differences in antibody titers were observed between cattle and buffalo (ns, *p* > 0.05), indicating comparable immune responses in both species. However, significant variations were noted between different time intervals postvaccination (*p* < 0.05), reflecting dynamic changes in antibody levels.

**Table 2:** Serum antibody titers measured by SAT in both cattle and buffalo calves following vaccination with *B. abortus* S19 (4 × 10^9^ CFU/dose) at different DPV intervals.

DPVs	*B. abortus* S19 in cattle and buffalo calves (4 × 10^9^ CFU/dose; *n* = 15)				
	< 80	1:80	1:160	1:320	1:640
0	15				
< 21					15
21–45				15	
46–60			15		
61–90		15			
91–120	15				
> 120	15				

Data are presented as mean ± SD.

*SAT*, standard agglutination test; *B. abortus S19*, *Brucella abortus* S19 strain vaccine; DPV, days of postvaccination; *CFU*, colony-forming units.

#### 3.2.3 Humoral immune response of IgG and IgM detection by iELISA

**Figure 2:**
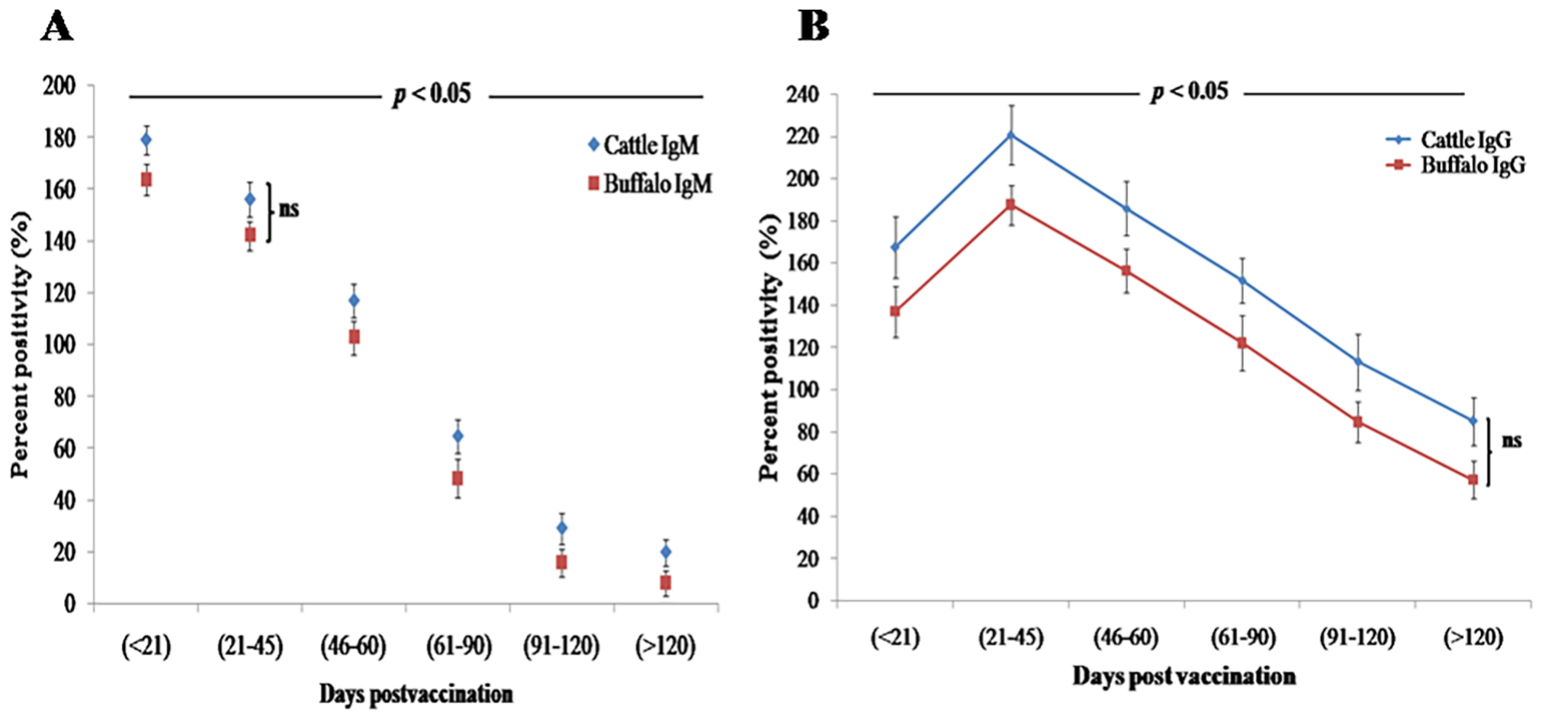
Humoral immune response kinetics in cattle and buffalo calves vaccinated with *B. abortus* S19 (4 × 10^9^ CFU/dose) at different DPV intervals, measured by iELISA. Data are presented as mean ± SD; **(A)** IgM and **(B)** IgG antibody levels.

Quantification of antibody concentrations by iELISA revealed a biphasic IgM response, with an initial statistically significant peak (*p* < 0.001) at DPV < 21 (cattle: 179.2 PP ± 5.5 PP; buffalo: 163.7 PP ± 5.9 PP), followed by a secondary peak at DPV 21–45 (cattle: 156.2 PP ± 6.8 PP; buffalo: 142.2 PP ± 5.5 PP). IgM levels declined significantly (*p* < 0.05) by DPV 46–60 in both species (**Figure 2A**). Concurrently, IgG seroconversion became statistically significant (*p* < 0.001) by DPV 21–45, reaching maximum PP values (cattle: 220.6 ± 13.9; buffalo: 187.5 ± 9.5) that were significantly higher (*p* < 0.01) than corresponding IgM levels (**Figure 2B**). While IgG remained detectable through DPV > 120 in both species, cattle exhibited significantly faster (*p* < 0.05) IgM-to-IgG class switching compared to buffaloes. Despite this difference in switching kinetics, no significant differences in overall antibody titers were observed between cattle and buffaloes at any time point (ns, *p* > 0.05), confirming comparable immune responses to *B. abortus* S19 vaccination. However, statistically significant variations (*p* < 0.05) between postvaccination intervals highlighted dynamic changes in antibody kinetics, underscoring the robust yet temporally distinct humoral immune activation in both species.

#### 3.2.4 Postvaccination innate immune responses

**Figure 3:**
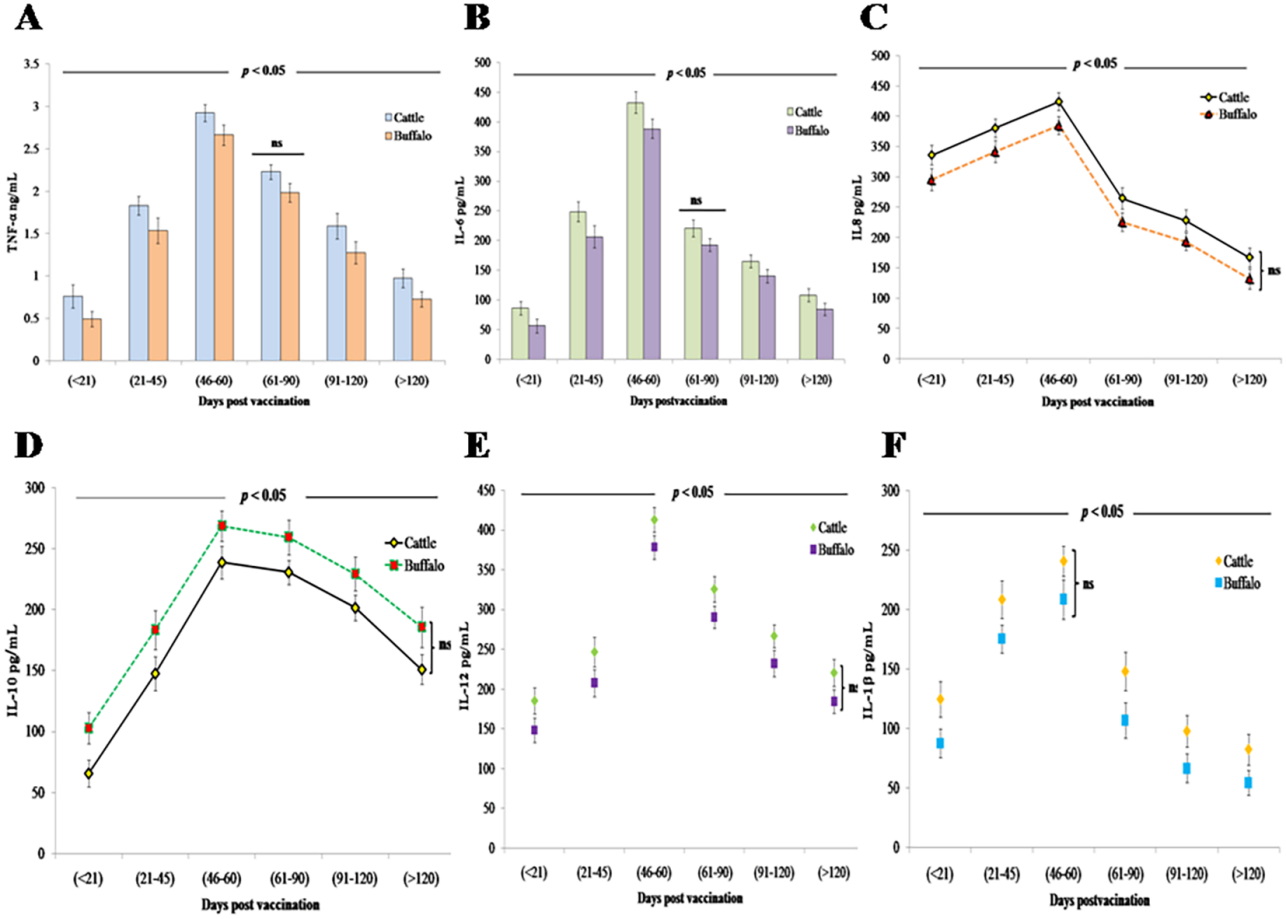
Innate immune cytokine responses in cattle and buffalo calves vaccinated with *B. abortus* S19 (4 × 10^9^ CFU/dose): Temporal profiles of **(A)** TNF-α, **(B)** IL-6 (pg/mL), **(C)** IL-8 (pg/mL), **(D)** IL-10 (pg/mL), **(E)** IL-12 (pg/mL), and **(F)** IL-1β (pg/mL) at different DPVs. Data represents mean cytokine levels (ng/mL or pg/ML) ± SD.

Vaccination with *B. abortus* S19 at 4 × 10^9^ CFU/dose triggered a significant (*p* < 0.05) inflammatory response in both cattle and buffalo calves, characterized by elevated levels of proinflammatory cytokines (TNF-α, IL-6, IL-8, IL-12, and IL-1β) along with the anti-inflammatory cytokine IL-10. TNF-α showed an early statistically significant increase (*p* < 0.01) at DPV < 21 (cattle: 0.761 ng/mL ± 0.14 ng/mL; buffalo: 0.493 ng/mL ± 0.09 ng/mL), reaching its peak (*p* < 0.001) at DPV 46–60 (cattle: 2.923 ng/mL ± 0.1 ng/mL; buffalo: 2.662 ng/mL ± 0.12 ng/mL) before declining significantly (*p* < 0.05) (**Figure 3A**). IL-6 demonstrated similar kinetics, with significant elevation (*p <* 0.01) at DPV < 21 (cattle: 86.2 pg/mL ± 11.6 pg/mL; buffalo: 56.8 pg/mL ± 11.8 pg/mL) that persisted through DPV 46–60 (cattle: 432.6 pg/mL ± 18.2 pg/mL; buffalo: 388.4 pg/mL ± 16.4 pg/mL) before decreasing significantly (*p* < 0.05) (**Figure 3B**). IL-8 showed its highest statistically significant levels (*p* < 0.001) at DPV 46–60 (cattle: 424.5 pg/mL ± 15.2 pg/mL; buffalo: 385.3 pg/mL ± 14.7 pg/mL), followed by a marked decline (*p* < 0.01) (**Figure 3C**).

The anti-inflammatory cytokine IL-10 exhibited its most pronounced regulatory effect at DPV 46–60 (cattle: 234.7 pg/mL ± 13.6 pg/mL; buffalo: 268.5 pg/mL ±12.4 pg/mL; *p* < 0.001), demonstrating a strong negative correlation with IFN-γ production in both species (**Figure 3D**). Buffalo calves exhibited higher levels compared to cattle, likely due to species-specific immune regulatory mechanisms that favor a stronger feedback inhibition of proinflammatory responses.

The concentrations of IL-12, a key Th1-polarizing cytokine, and IL-1β, an important proinflammatory mediator, exhibited significant temporal dynamics following vaccination. IL-12 responses peaked (*p* < 0.001) at DPV 46–60 in both cattle (412.7 pg/mL ± 15.3 pg/mL) and buffalo (378.4 pg/mL ± 14.8 pg/mL) (**Figure 3E**). Similarly, IL-1β concentrations in cattle reached their maximum levels (238.6 pg/mL ± 16.5 pg/mL; *p* < 0.001) during the same DPV interval (46–60) before subsequently declining. This coordinated peak of key immune mediators suggests a synchronized proinflammatory response following vaccination in cattle. However, a comparable vaccine-induced IL-1β response was not observed in buffalo calves (**Figure 3F**). Despite these trends, no statistically significant differences in cytokine levels were observed between cattle and buffalo at any specific time point (ns, *p* > 0.05), although significant variations were noted across different postvaccination intervals (*p* < 0.05), reflecting dynamic cytokine responses over time.

### 3.3 CMI response to *B. abortus* S19 vaccination

**Figure 4:**
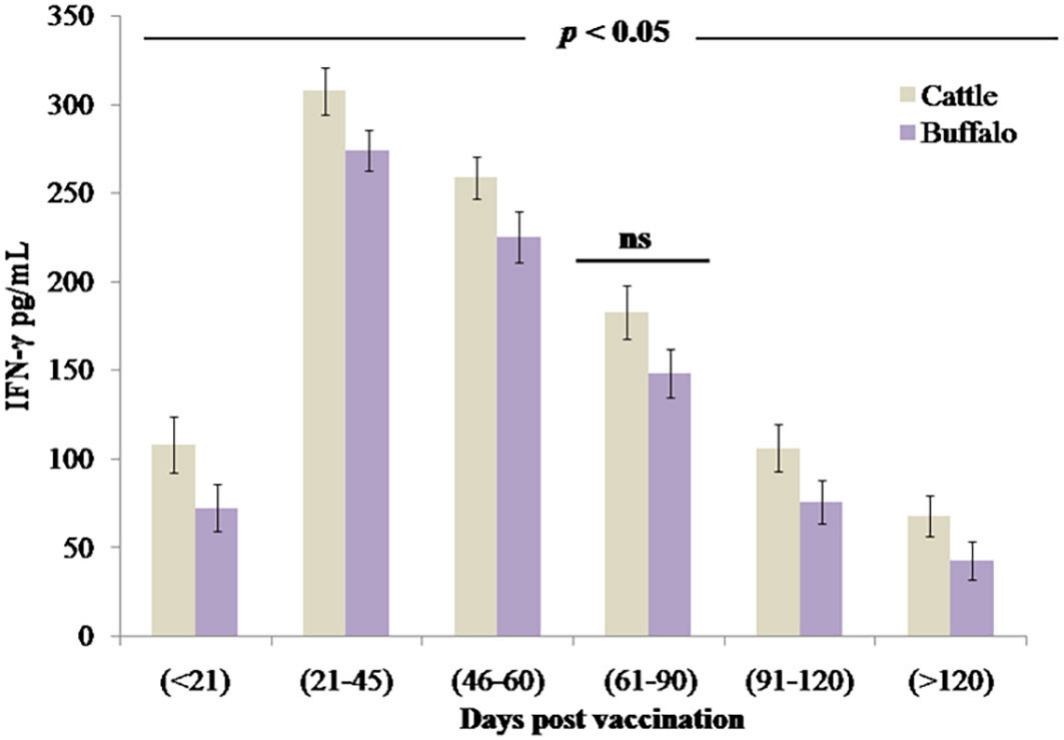
The cell-mediated immune response measured through IFN-γ production (pg/mL) in cattle and buffalo calves vaccinated with *B. abortus* S19 (4 × 10^9^ CFU/dose) at different DPVs. Data represent mean cytokine levels (pg/mL) ± SD.

The study quantified IFN-γ, a key mediator of cell-mediated immunity and macrophage activation. Vaccination with *B. abortus* S19 induced a robust Th1-type immune response in both cattle and buffalo calves, demonstrated by significant IFN-γ production (*p* < 0.001) as early as DPV < 21 (cattle: 108.4 pg/mL ± 15.8 pg/mL; buffalo: 72.7 pg/mL ± 13.4 pg/mL). IFN-γ levels peaked at DPV 21–45 in both cattle (307.7 pg/mL ± 13.4 pg/mL) and buffalo (274.2 pg/mL ± 11.7 pg/mL), followed by a gradual but significant decline (*p* < 0.05 at each interval) (**Figure 4**). Analysis revealed that the 4 × 10^9^ CFU/dose of *B. abortus* S19 induced a comparable magnitude and kinetics of IFN-γ response (*p* > 0.05) to conventional vaccine doses, confirming its efficacy in stimulating protective cell-mediated immunity. No statistically significant differences were observed between cattle and buffalo at any time point (ns, *p* > 0.05); however, significant variations across postvaccination intervals (*p* < 0.05) demonstrated dynamic patterns of immune activation over time.

## 4 Discussion

Brucellosis is a significant zoonotic disease affecting livestock, caused by *B. abortus.* The S19 vaccine has been widely used to prevent brucellosis in livestock; however, challenges related to cost, vaccine safety, and efficacy continue to limit its optimal use, especially in resource-limited settings. The present study comprehensively evaluated the immunogenicity, safety, and cytokine response dynamics in cattle and buffalo calves following vaccination with *B. abortus* S19 at a dose of 4 × 10^9^ CFU/dose in naturally reared calves. The findings demonstrate that this dose maintains a favorable safety profile. Importantly, no adverse local or systemic reactions were observed in any vaccinated animals during the 3-week postvaccination monitoring period, confirming the vaccine’s excellent tolerability at the tested dose under field conditions. This safety profile is consistent with or superior to conventional doses reported in earlier studies, supporting the appropriateness of 4 × 10^9^ CFU/dose as a viable vaccination strategy. Consistent with previous reports in cattle (17–19) and buffalo (20–22), our findings demonstrate that *B. abortus* S19, administered at 4 × 10^9^ CFU/dose, maintains a validated safety profile.

Serological assessments using RBPT and SAT revealed statistically significant antibody responses (*p* < 0.05) in both cattle and buffalo, peaking between 21 and 45 DPV and persisting beyond 120 days. These results align with previous studies showing that both full (40 × 10^10^ CFU/dose) and reduced (4 × 10^9^ CFU/dose) doses of *B. abortus* S19 sustain serological responses up to 240 DPV (15, 16). RBPT results confirm that the *B. abortus* S19 at 4 × 10^9^ CFU/dose effectively induces durable humoral immunity comparable to conventional doses reported in other studies for both bovine species. SAT titers remained ≥ 80 IU/mL through DPV > 120, confirming durable humoral immunity and indicating that lower antigenic loads can elicit long-lasting antibody levels. Vaccinated animals developed high anti-LPS IgM and IgG titers but showed minimal immune responses to cytoplasmic proteins, suggesting the potential utility of these markers for differential diagnosis (23). Additionally, multiple studies have collectively shown that reduced doses of the *B. abortus* S19 vaccine can effectively induce robust immune responses in both cattle and buffaloes (24). Our iELISA analysis further clarified the kinetics of immunoglobulin responses, revealing a biphasic IgM response followed by significant IgG seroconversion, consistent with established immunological patterns. Notably, the 4 × 10^9^ CFU/dose induced IgG levels comparable to those elicited by the conventional 40 × 10^10^ CFU/dose (*p* > 0.05), supporting a dose-efficacy paradigm in which lower antigen loads sufficiently prime adaptive immunity while reducing biological waste. A novel finding from this study is the significantly faster IgM-to-IgG class switching in cattle compared to buffalo (*p* < 0.05), suggesting species-specific differences in germinal center dynamics and B-cell maturation. Importantly, no significant differences in overall antibody titers were observed between cattle and buffalo at any time point (ns, *p* > 0.05), confirming comparable humoral immune responses to *B. abortus* S19 vaccination. However, statistically significant variations between postvaccination intervals (*p* < 0.05) reflected dynamic changes in antibody kinetics, underscoring robust yet temporally distinct humoral immune activation in both species.

Cytokine profiling revealed significant temporal variations in both proinflammatory and anti-inflammatory mediators following vaccination with *B. abortus* S19 at a dose of 4 × 10^9^ CFU. Recent studies comparing immune responses elicited by full (4 × 10^10^ CFU/dose) and reduced (4 × 10^9^ CFU/dose) vaccine doses demonstrated elevated levels of key inflammatory markers, including TNF-α, IL-6, IL-12, and IFN-γ, in both regimens. These findings indicate cellular immune activation occurs even at the reduced 4 × 10^9^ CFU/dose reduction (16, 25). In our study, elevated levels of proinflammatory cytokines (TNF-α, IL-6, IL-8, IL-12, and IL-1β) were observed, peaking between 46 and 60 DPV. This inflammatory response was accompanied by a concurrent increase in the anti-inflammatory cytokine IL-10, suggesting a transition from immune activation to regulation. These findings align with prior reports demonstrating that early IL-8 upregulation reflects acute innate immune activation, whereas delayed IL-10 expression coincides with the resolution phase (25–27).

The strong negative correlation observed between IL-10 and IFN-γ levels highlights a critical immunoregulatory mechanism that tempers excessive inflammation while maintaining protective immunity. Elevated IL-12 and IL-1β levels further support the activation of dendritic cells and macrophages, key components of the Th1-polarized immune response. Although transient IL-1β elevations have been associated with inflammatory pathology (28), our data indicate that this response was effectively self-limited without adverse effects. Importantly, a robust IFN-γ response, a hallmark of protective Th1-type immunity, was detected, peaking at DPV 21–45 and remaining sustained thereafter. This pattern is consistent with cyclic IFN-γ trends reported around DPV 21, 60, and 120, suggesting strategic time points for booster administration (29). While no statistically significant differences in cytokine expression were observed between cattle and buffalo at any time point (ns, *p* > 0.05), significant variations across postvaccination intervals (*p* < 0.05) reflect dynamic, time-dependent immune activation. These findings demonstrate that *B. abortus* S19 at 4 × 10^9^ CFU/dose effectively induces durable Th1-type cell-mediated immunity, providing robust and comparable protection against brucellosis in both cattle and buffalo (30). The divergence in cytokine profiles suggests that cattle rely on a robust inflammatory response for pathogen clearance, whereas buffalo prioritize immune regulation to mitigate collateral damage, highlighting fundamental differences in their immunological strategies. The higher levels of proinflammatory cytokines—TNF-α, IL-6, IL-8, IL-12, and IL-1β—observed in cattle indicate a stronger proinflammatory response. While this may enhance early pathogen clearance, it could also lead to prolonged inflammation and potential immunopathology, highlighting a key immunological difference between the two species in balancing immune activation and regulation.

Finally, our study successfully validated the safety and immunogenicity of *B. abortus* S19 at a dose of 4 × 10^9^ CFU in cattle and buffalo calves, addressing key objectives. First, the absence of local or systemic adverse effects confirmed an excellent safety profile, consistent with historical data for S19. Second, early immune biomarkers—IgM, IL-8, and IL-1β—peaked at DPV < 21, serving as predictive indicators of vaccine uptake and initial inflammatory responses. Third, durable immunity was demonstrated through sustained IgG (SAT/RBPT/iELISA) and IFN-γ responses persisting beyond DPV 120, albeit with gradual waning, aligning with protective efficacy timelines reported for conventional doses. Notably, the 4 × 10^9^ CFU dose elicited humoral and cellular responses comparable to higher doses, supporting its potential as a cost-effective alternative. Fourth, species-specific differences emerged: cattle exhibited faster IgM-to-IgG switching and higher IFN-γ production than buffalo, suggesting divergent B-cell and Th1 response kinetics. Lastly, the coordinated proinflammatory (TNF-α, IL-6, IL-12) and anti-inflammatory (IL-10) cytokine dynamics highlighted robust immune regulation postvaccination. Collectively, our data support the use of the *B. abortus* S19 vaccine at 4 × 10^9^ CFU/dose vaccine to achieve effective, species-informed brucellosis control while minimizing antigen usage and associated biological waste.

The follow-up period in this study was limited to over 120 DPV, shorter than the duration of immune responses reported in earlier studies. Previous research has shown that both serological titers and CMI responses can persist for up to 240 days following *B. abortus* S19 vaccination (15). Although our results demonstrated robust and durable immunity, as indicated by sustained IgG titers, cytokine activation, and IFN-γ production throughout the 120-day period, long-term monitoring was not included in the current study design. Earlier studies, including those by Chaithra et al. (29), reported that both conventional (4 × 10^10^ CFU) and reduced (4 × 10^9^ CFU) doses of S19 can maintain detectable antibody and cellular responses well beyond 200 days, suggesting potential for extended protection. Therefore, while our findings confirm strong immunogenicity of the 4 × 10^9^ CFU dose up to 120 days, longitudinal studies are needed to validate response persistence beyond this time frame and to align with the ~ 240-day protection duration reported in the literature. The study also identified species-specific immune response patterns: cattle exhibited higher levels of IFN-γ, a hallmark of Th1-mediated CMI, whereas buffaloes showed elevated IL-10, indicating a stronger regulatory and anti-inflammatory response. These findings are consistent with earlier reports highlighting fundamental immunological differences between cattle and buffalo, where cattle mount more robust Th1-polarized responses, and buffaloes often exhibit delayed IgG class switching and increased immune regulation (13). Elevated IFN-γ in cattle may contribute to faster bacterial clearance, while higher IL-10 levels in buffaloes could serve as a compensatory mechanism to control inflammation, potentially at the expense of effective pathogen clearance.

Although our study documents these immunological differences, the underlying mechanisms and their implications for long-term protection remain unclear. Future studies incorporating transcriptomic analysis or immune cell profiling are needed to determine whether these divergent responses reflect adaptive evolutionary strategies or potential limitations in vaccine efficacy across species (30). Our data demonstrated strong humoral (IgM/IgG), innate (cytokines), and CMI (IFN-γ) responses, all established correlates of protective immunity in brucellosis (30). While we did not directly measure abortion rates or transmission, the sustained IgG levels and Th1-type responses observed are consistent with markers associated with reduced bacterial persistence and enhanced protection (29). In future research, we plan to integrate immunological assessments with reproductive outcomes and transmission dynamics to establish stronger correlations between laboratory markers and protection under field conditions.

These species-specific immune profiles also have practical implications for vaccination strategies. Cattle, which mounted stronger Th1 responses (higher IFN-γ and faster IgG switching), may achieve more efficient bacterial clearance, whereas buffaloes, characterized by elevated IL-10 and delayed IgG seroconversion, may exhibit weaker protective responses (15, 27, 31). This underscores the need for tailored vaccination approaches, such as booster strategies for buffaloes, to optimize protection and enhance the effectiveness of brucellosis control programs. A logical extension of this study would be to conduct follow-up trials extending beyond 240 days to assess the durability of immune responses and protective efficacy under field conditions. Long-term evaluation is critical, as waning antibody levels or cellular immunity could compromise vaccine effectiveness and influence the success of eradication efforts (32). Extended monitoring would enable detailed analysis of memory B- and T-cell responses, serological persistence, and booster requirements, all of which are essential for designing sustainable control strategies (33).

Furthermore, studies across varied management systems and ecological settings would enhance our understanding of vaccine performance under real-world conditions. Serological interference caused by S19 vaccination remains a major challenge in brucellosis surveillance. Vaccine-induced antibodies can cross-react with standard diagnostic tests (RBPT, SAT, ELISA), complicating the differentiation between infected and vaccinated animals. This may result in false-positive diagnosis, inaccurate herd prevalence estimates, unnecessary culling, and reduced farmer compliance. To address these issues, it is essential to adopt differentiating infected from vaccinated animals (DIVA) strategies, including molecular diagnostics, epitope-specific assays, or advanced platforms such as the fluorescence polarization assay (FPA) (34). Integrating these refined tools with conventional serological methods will improve diagnostic specificity, enhance surveillance accuracy, and support more effective eradication programs.

We acknowledge that the present study provides only a limited discussion on the issue of serodiagnostic interference, which remains a critical consideration for the use of *B. abortus* S19 in eradication programs. A well-documented limitation of S19 vaccination is the production of antibodies against the sLPS antigen from *B. abortus* S99, which cannot be reliably distinguished from those generated during natural infection using conventional tests such as RBPT and SAT (35). This diagnostic interference poses challenges for test-and-slaughter strategies central to eradication efforts. However, reduced-dose formulations, including the 4 × 10^9^ CFU used in this study, have been shown to elicit robust immunity while minimizing the duration of seropositivity, thereby potentially reducing interference compared with conventional doses (16). Advances such as differential diagnostic assays targeting cytoplasmic proteins or recombinant antigens offer additional promise for distinguishing vaccinated from infected animals (1). While our study primarily focused on vaccine safety and immunogenicity, we recognize that addressing serodiagnostic interference is vital for the integration of S19 vaccination into national control programs, and future work should evaluate how modified-dose vaccination aligns with DIVA strategies.

## 5 Conclusions

In conclusion, this comprehensive study clearly demonstrates that the *B. abortus* S19 vaccine formulation, 4 × 10^9^ CFU/dose, elicited strong and durable humoral, innate, and CMI responses without any observed adverse effects in cattle and buffalo calves. The antibody production (IgM and IgG), sustained seroconversion detected via RBPT, SAT, and iELISA, along with significant IFN-γ induction, a hallmark of Th1-type immunity, confirmed its immunogenicity. The identification of early immune biomarkers, such as IgM, IL-8, and IL-1β, and cytokine profiling revealed coordinated activation of pro- and anti-inflammatory mediators, with cattle exhibiting a more pronounced proinflammatory response than buffaloes, demonstrating stronger regulatory control through elevated IL-10 levels. These species-specific immune dynamics, despite differing response patterns, resulted in comparable levels of protection. Importantly, no statistically significant differences were observed between species in the overall immune response magnitude. These findings not only validate the 4 × 10^9^ CFU dose as a scientifically robust and cost-effective alternative to conventional formulations but also provide crucial insights for optimizing vaccination protocols, particularly addressing the previously demonstrated experimental immune responses in buffalo calves. Overall, the 4 × 10^9^ CFU/dose of *B. abortus* S19 demonstrates excellent safety, strong immunogenicity, and effective protection, supporting its use as a standardized, practical tool for brucellosis control in bovine populations.

## Data Availability

The original contributions presented in the study are included in the article/supplementary material. Further inquiries can be directed to the corresponding author.
